# Uncovering the hidden structure of small-world networks

**DOI:** 10.1038/s41598-023-50651-x

**Published:** 2024-03-19

**Authors:** Ahmed Lachgar, Abdelfattah Achahbar

**Affiliations:** Condensed Matter Group, Department of Physics, Faculty of Sciences, Abdelmalek Essâadi University, Tétouan, Morocco

**Keywords:** Physics, Statistical physics, thermodynamics and nonlinear dynamics

## Abstract

The small-world (SW) network model introduced by Watts and Strogatz has significantly influenced the study of complex systems, spurring the development of network science as an interdisciplinary field. The Newman-Watts model is widely applied to analyze SW networks by adding several randomly placed shortcuts to a regular lattice. We meticulously examine related previous works and conclude that the scaling of various pertinent quantities lacks convincing evidence. We demonstrate that the SW property primarily stems from the existence of clusters of nodes linked by shortcuts rather than just the mean number of shortcuts. Introducing the mean degree of clusters linked by shortcuts as a new key parameter resolves the scaling ambiguity, yielding a more precise characterization of the network. Our findings provide a new framework for analyzing SW networks, highlighting the significance of considering emergent structures in complex systems. We also develop a phase diagram of the crossover transition from the small to the large world, offering profound insights into the nature of complex networks and highlighting the power of emergence in shaping their behavior.

## Introduction

Complex networks are integral to various scientific disciplines, impacting fields as diverse as social, biological, transportation, and communication networks^1–7^. Among the intriguing aspects of these networks is the presence of small-world (SW) behavior, characterized by short path lengths between nodes. The Newman-Watts (NW) SW model stands out as a notably well-established and thoroughly researched paradigm within such networks^8–14^. This model is distinguished by the addition of new connections to an existing network based on a predetermined probability. These additional links, known as shortcuts, introduce a mix of order and randomness to the model, reflecting social patterns where individuals are more closely connected to those in their immediate vicinity than to those farther away.

A fundamental metric for the characterization of SW networks is the mean distance, denoted as $\ell$, which measures the average distance between nodes. This metric provides valuable insights into the network’s inherent properties, allowing us to discern whether it exhibits SW behavior, characterized by $\ell \sim \ln (n)$, or large-world behavior, with $\ell \sim n$. The scaling relationship for $\ell$, as originally formulated by Newman and Watts using a renormalization group transformation (RG), can be expressed as $\ell = \frac{n}{k}f(nk\phi )$. Here, *n* denotes the total number of nodes, *k* represents the regular degree of the network, and $\phi$ quantifies the probability of introducing shortcuts between pairs of nodes^9^. Several critical issues must be addressed in the context of scaling analyses applied to SW networks. First, scaling is only valid when the mean degree of clusters due to shortcuts is very small, which occurs when $k^2\phi \ll 1$^9^, where the network is almost regular and does not exhibit SW characteristics. Second, when studying the parameter $n^*$, which represents the network size at which it becomes SW, it is expected to behave as $n\sim \phi ^{-\tau }$ with $\tau =1$^9,15,16^. However, no universal behavior is observed for $n^*$ with $\phi$, and the data collapse is valid only for each value of the degree *k*. This is problematic because the degree of the network is one of the arguments of the universal function, which requires the universal behavior of all system magnitudes according to *k*. Moreover, the mean field solution for the scaling function given by Newman et al.^10^, with the mean number of shortcuts $x=nk\phi$ as the relevant parameter, is only exact for small and large values of *x*. The solution fails when the probability $\phi$ is close to one, as there is a clear difference between the simulations and the mean-field solution, even for smaller values of $\phi$. Thus, the scaling function that is often used to refer to the mean distance in SW networks is inadequate, and data collapse does not necessarily prove that the mean distance follows this path. Instead, this scaling function is more appropriate, as we will show, for representing the mean distance in regular networks. In our study, we introduce an innovative method to reveal the underlying structure of SW networks. Our approach first employs RG techniques, followed by a strategic division of the network into two distinct subnetworks. One subnetwork consists of regular nodes, while the other encompasses random nodes influenced by shortcuts. This bifurcation enables us to meticulously analyze the behavior of each subnetwork, facilitating a more accurate depiction of the scaling behavior of $\ell$. Our research indicates that these networks exhibit a sophisticated pattern of organization that results from the appearance and cooperation of groups of nodes. However, this structure cannot be identified using traditional network analysis methods that focus on individual nodes.

## Mean distance

The mean distance in a circular network is $\nonumber \langle \hat{\ell } \rangle =\sum _{\hat{\ell }=1}^{\frac{\hat{n}}{2}}\hat{\ell }\cdot n(\hat{\ell })\approx \int _1^{\frac{{\hat{n}}}{2}} \hat{\ell }\cdot n(\hat{\ell }) \cdot d\hat{\ell }$, where $n(\hat{\ell })$ is the number of nodes at a distance $\hat{\ell }$ from a given node. Initially, we use the RG method and subsequently divide the network into two sub-networks. The first sub-network is composed of nodes that are not impacted by shortcuts and we refer to them as “regular nodes”. The second sub-network is made up of nodes that are influenced by shortcuts, which we term “random nodes”. With this approach, we compute the mean distance of the network and obtain the corresponding formula outlined in Methods:

1 $$\begin{aligned} \langle \hat{\ell } \rangle =\left (\frac{W\bigg (\frac{\left( \ln (y+1)\right) ^2(y+1)}{4p}\bigg )}{\ln (y+1)}+1\right )h(x)+\hat{n}\frac{1-e^{-x}}{4x}, \end{aligned}$$

where $x=nk\phi$ is the mean number of shortcuts, and $y=2k^2\phi$ represents the mean degree of clusters linked by shortcuts (see Methods) .

As the value of *x* approaches 0, the network becomes more regular and $\lim \limits _{x \rightarrow 0}h(x)=0$. In this scenario, the mean distance of the network approaches $\frac{n}{4k}$, thereby validating our calculation. Indeed, with periodic boundary conditions on regular circular network $\langle \hat{\ell }\rangle =\frac{{n}}{4k}$.

**Figure 1:**
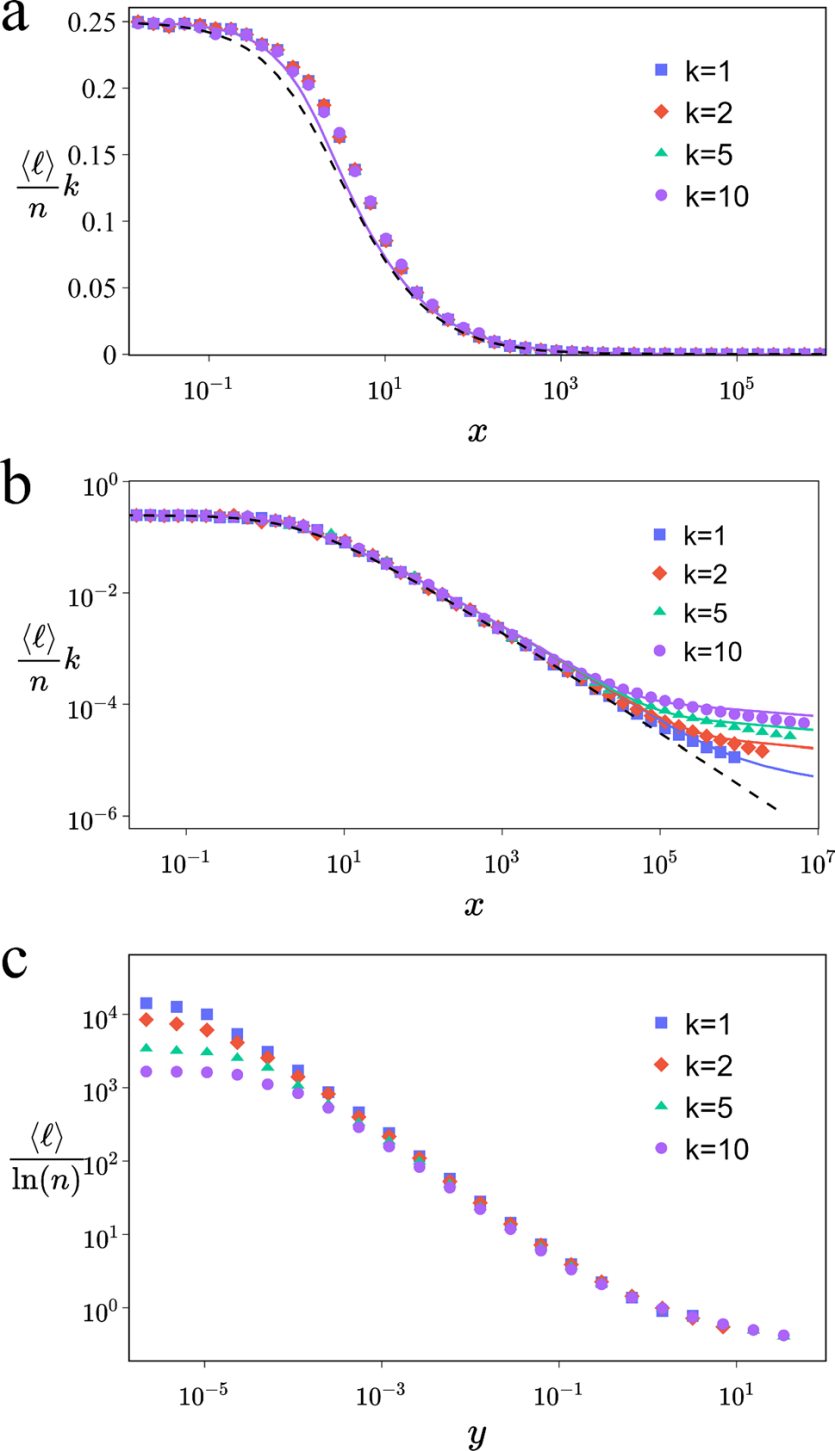
Scaling of the mean distance. (**a**,**b**), Behavior of $\frac{\langle {\ell }\rangle }{n}k$ as a function of *x* for various values of *k* (from top to bottom $k=10,5,2,1$), network size is $n=10^6$. Each simulation is averaged over 100 achievements. In both figures (1) is the continuous line, (2) is the dashed line. The scale is semi-logarithmic in (**a**) and log-log in (**b**). (**c**) $\frac{\langle \ell \rangle }{\ln (n)}$ as a function of *y* for various values of *k* (from top to bottom $k=1,2,5,10$), $n=10^6$, number of realizations for each simulations is 50. The scale is logarithmic.

Figure 1a and b serve as robust validations of our methodology, providing compelling evidence for our approach. They distinctly illustrate the alignment between simulation results and (1). Notably, (1) outperforms the Newman et al. Eq. (2) in accuracy^10^. Yet, we note discrepancies in simulations occurring within the same region as highlighted by Newman et al.

For large-world networks where $\langle {\ell }\rangle \propto n$, the NW scaling function appears to be universal for small values of *x*. However, Newman et al. have shown that their mean field solution (2) breaks down as the density of shortcuts increases^10^:

2 $$\begin{aligned} f(x)=\frac{1}{2\sqrt{x^2+2x}}\tanh ^{-1}\left(\sqrt{\frac{x}{x+2}}\right). \end{aligned}$$

**Figure 2:**
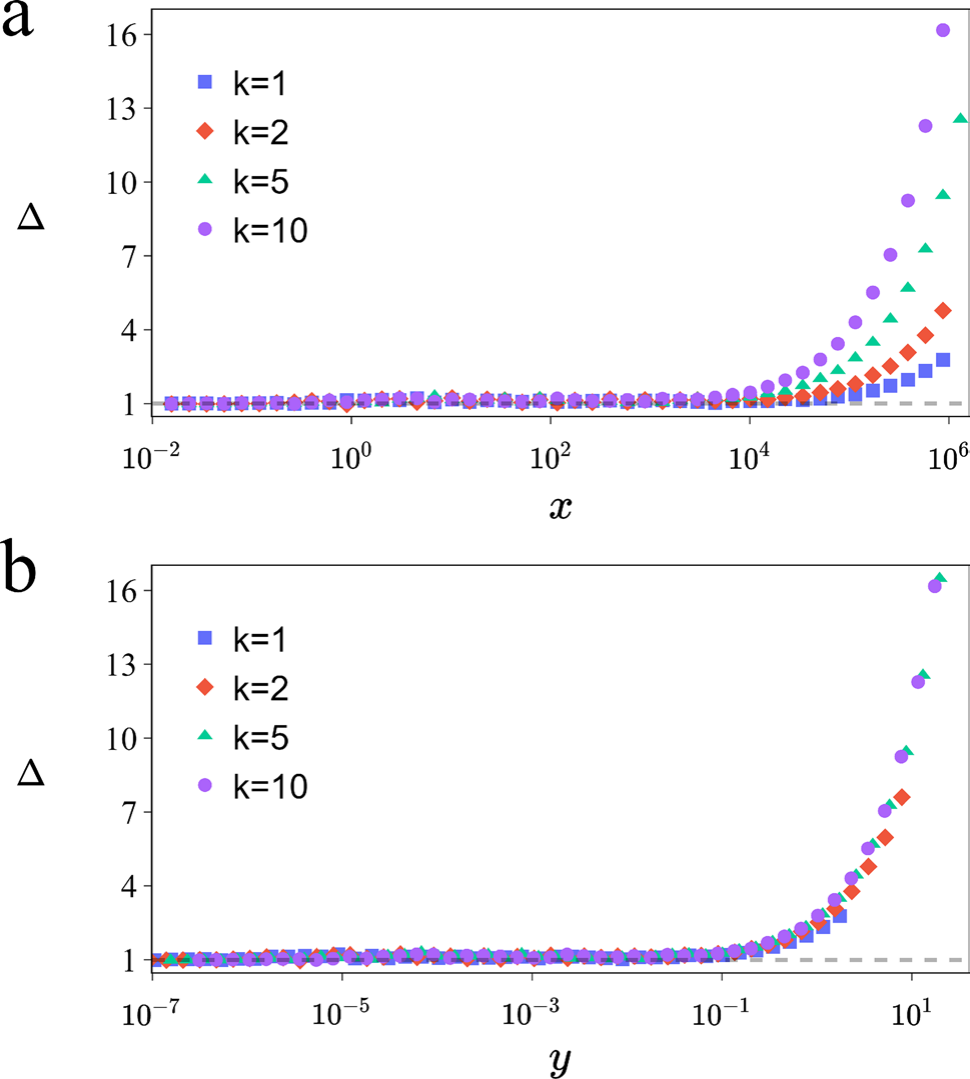
Validity of the universal function *f*(*x*). Variations of $\Delta$ with *x* in (**a**) and with *y* in (**b**) for various values of *k* (from top to bottom $k=10,5,2,1$), $n=10^6$. The number of achievements for each simulation is 1000. The scale is semi-logarithmic.

Figures 1b and 2a present compelling simulation results that challenge the notion of universality in large *x* values, particularly in the region traditionally classified as SW. These figures clearly depict systematic deviations for varying values of *k*, thereby undermining the assumption of universality in this regime. This observation is critical as it suggests that the mean distance within SW networks is influenced by factors beyond just the mean number of shortcuts, denoted by *x*. Our analysis points towards the need to consider an alternative parameter to more accurately characterize the dynamics of SW networks. Upon the application of the RG transformation, we define *y* as the mean degree of clusters interconnected by shortcuts. This parameter finds a parallel in the Erdös-Rényi network model, where *y* corresponds to the average degree of nodes. In this analogy, the clusters in our model are akin to nodes, and the shortcuts to links in the Erdös-Rényi framework. Notably, *y* emerges naturally in the random component of the mean distance expression, as delineated in Eq. (1). Given its fundamental role in this crucial expression and its similarity to the Erdős-Rényi network, *y* will undergo meticulous evaluation to ascertain whether it truly serves as the central parameter controlling the dynamics of SW networks.

If *y* is significantly small, the network behaves like a large-world network and does not exhibit the SW property. Taking $y\ll 1$ in (1) we get:

3 $$\begin{aligned} \langle \hat{\ell } \rangle =\hat{n}\frac{2W\left(\frac{x}{2}\right)h(x)+1-e^{-x}}{4x}. \end{aligned}$$

The expression for $\langle \hat{\ell } \rangle$ in (3) does not directly involve *y*, suggesting that the appropriate scaling parameter in this regime may be *x*. On the other hand, if we assume that *n* is large and *y* is not too small in (1), we can approximate $W\big (\frac{\ln (y+1)^2(y+1)}{4p}\big )$ as $\ln \big (\frac{\ln (y+1)^2(y+1)}{4p}\big )$, *h*(*x*) approaches 1, and $\frac{y+1}{4p}=n\frac{2k^2\phi +1}{8k^3\phi }$. Under these conditions, (1) becomes:

4 $$\begin{aligned} \langle {{\hat{\ell }}} \rangle= & {} \frac{\ln \ln (y+1)^2+\ln \frac{y+1}{4p}}{\ln (y+1)}+1+\frac{1-e^{-x}}{2y} \nonumber \\\approx & {} \frac{\ln n}{\ln (y+1)}. \end{aligned}$$

The above equation that displays the SW phenomenon is identical to what is observed in the Erdös-Rényi model^17^. Consequently, the appropriate universal function that applies in this context is:

5 $$\begin{aligned} g(y)=\frac{1}{\ln (y+1)}. \end{aligned}$$

A critical insight from our study is that the mean degree of clusters interconnected by shortcuts emerges as the sole relevant scaling parameter, a conclusion vividly illustrated in Figs. 1 and 2. This observation underscores the pivotal role of these inter-cluster connections in defining the network’s characteristics.

To substantiate our insights regarding the parameters $x$ and $y$, we introduce a new parameter, $\Delta$, defined as $\Delta = \frac{\frac{\langle \ell \rangle }{\hat{n}}}{f(x)}$. If $f(x)$ truly represents a universal function, then $\Delta$ should ideally equal 1. In Fig. 2, we plot $\Delta$ as a function of both $y$ and $x$, where $\langle \ell \rangle$ is derived from simulations of the Newman-Watts (NW) model, and $f(x)$ is given by (2). Our results reveal that $\Delta$ approximates 1 when $y$ is much less than 1. However, for larger values of $y$, $\Delta$ significantly exceeds 1, indicating the inadequacy of $f(x)$ under these conditions. This observation aligns with Newman’s earlier critique regarding the limitations of $f(x)$ as $\phi$ nears 1^10^.

Further, Fig. 2b illustrates that $f(x)$ loses its universality beyond certain $x$ values, which vary according to the network parameters. Consequently, this leads us to conclude that $y$, rather than $x$, functions as the actual control parameter in the NW model. Moreover, Fig. 1c provides compelling evidence supporting the significance of $y$ in the NW network. It demonstrates that in the large world regime, a small $y$ value fails to produce a data collapse with $\ln (n)$, suggesting that this range is not adequately described by $y$. In contrast, when $y$ is not exceedingly small, a data collapse is observed, affirming $y$ as an accurate descriptor of the system’s behavior. This finding not only corroborates our calculations but also significantly bolsters the validity of Eq. (4).

Another significant aspect is the necessity to express the system’s extensive quantities in terms of $\ln (n)$. This adjustment is crucial due to the SW property of the network, which creates a perception as if there are only $\ln (n)$ nodes present. This phenomenon leads to a conceptual shift from considering individual node identities to focusing on clusters. Such a perspective is instrumental in understanding and quantifying the emergence of SW behavior in these networks. By accounting for this, we can more accurately characterize the network dynamics and better understand the underpinnings of the SW phenomenon.

## Transition from large to small-world

**Figure 3:**
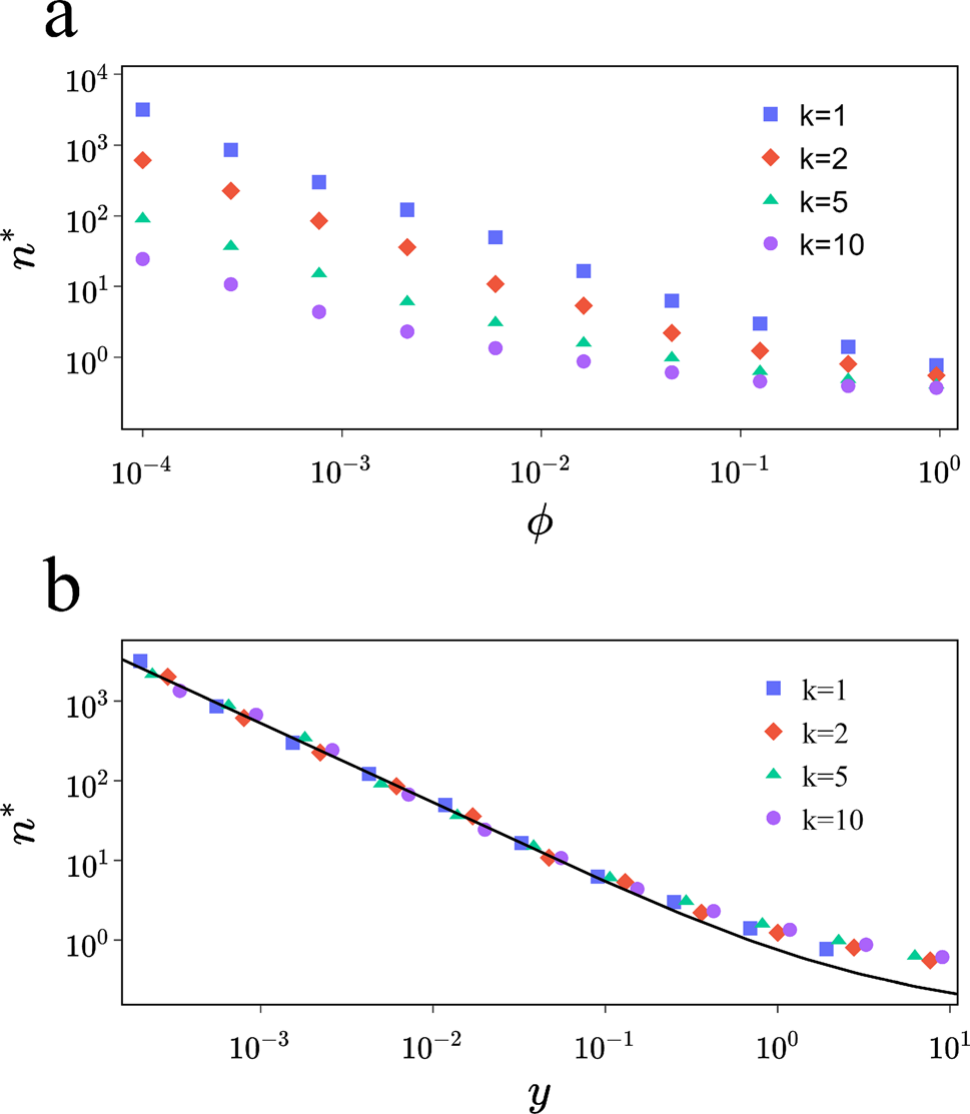
Data collapse and scaling of $n^{*}$. Scaling of $n^{*}$ as a function of $\phi$ (**a**) and as a function of *y* (**b**) for various values of *k* (from top to bottom $k=1,2,5,10$). The black line represents (5) multiplied by a constant. Each point is determined from the slope of $\ell$ as a function of $\ln (n)$. System size varies from 1000 to 200000, the number of realizations is 300 and the scale is logarithmic.

**Figure 4:**
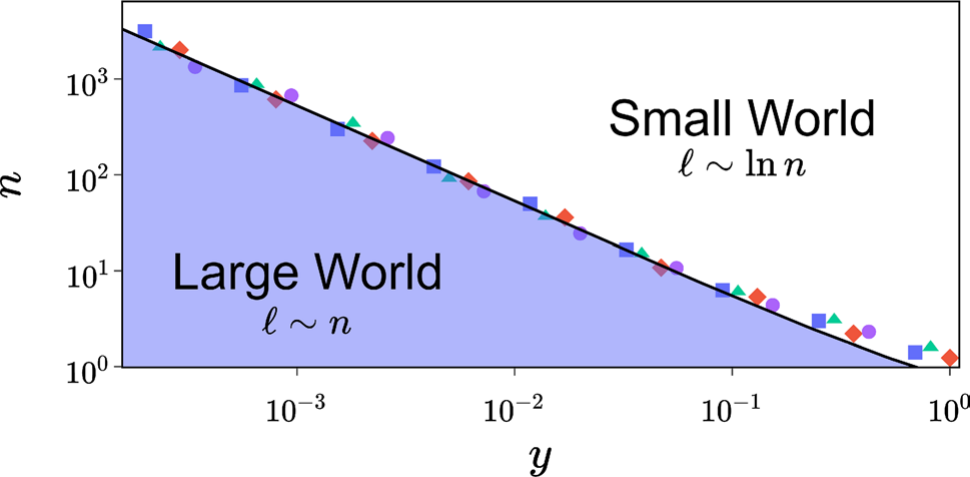
Phase Diagram of the SW network. Symbols (same as in Fig.3) are at the borders where $n=n^*$, i.e, the limits where the network passes from the large world ($\ell \sim n$) to the SW ($\ell \sim \ln \,n$).

Scaling analysis of $\langle \ell \rangle$ is carried out by introducing the parameter $n^{*}$, which represents the size of the network when it passes from large to SW. The scaling law for $\langle \ell \rangle$ can be written as^15^:

6 $$\begin{aligned} \langle \ell (n,\phi ,k)\rangle \sim n^{*}F\left(\frac{n}{n^{*}}\right), \end{aligned}$$

with $F(i\ll 1)\sim i$ and $F(i\gg 1)\sim \ln (i)$, hence $\ell (n\gg n^{*})\sim n^{*}\ln (n)$. Performing extensive simulations, we determine $n^{*}$ by calculating the slope of the curve of $\langle \ell \rangle$ as a function of $\ln n$. Simulations are repeated for several values of $\phi$ allowing to represent $n^{*}$ as a function of $\phi$ (see Fig. 3). Previously, it was believed that the value of $n^{*}$ is inversely proportional to $\phi$ raised to the power of $\tau =1$, as indicated in prior works such as those referenced in^9,15,16^. The data presented in Fig. 3a suggests that the relation between $n^{}$ and $\phi$ is not universal, as it is contingent on the values of *k*. However, based on (4) and (6), we can predict that if there exists a universal function of $n^{}$, it must be proportional to $g(y)=\frac{1}{\ln (y+1)}$. This hypothesis is strongly supported by the findings in Fig. 3b, which show excellent agreement between simulations and *g*(*y*). Furthermore, for the SW regime, it is deduced from these findings that $n^{*}\sim y^{-\tau }$, where $\tau =1$. The way $n^{*}$ behaves with respect to *y* excludes the possibility of a phase transition for all non-zero values of *y*, which supports the existence of a crossover region between the SW and large-world regimes^16^. Foreseeing whether a network is a small or large world is a vital element of this system. Our computations have unveiled the phase diagram of the transition, which empowers us to anticipate the nature of the network based on its parameters, and as a result, determine the transition line that distinguishes between the two regions (Fig. 4). Using the average number of shortcuts as a system control parameter would not have been viable because, as mentioned earlier, $n^{*}$ does not scale with it.

## Conclusion

In this study, we have employed a novel application of the RG transformation to dissect the complex structure of SW networks. Our approach distinctively categorizes nodes into ’regular’ and ’random’, unveiling the hidden architecture of these networks. This method underscores the concept of emergent behavior in SW networks, highlighting that the network’s macroscopic properties are not merely a sum of individual nodal connections, but rather a result of intricate interactions between clusters of nodes. Our findings suggest a significant reinterpretation of the SW regime, as previously defined in the NW model. We contend that this regime might more appropriately be characterized as a large-world regime. This reevaluation stems from our analysis showing that the average number of shortcuts, traditionally used as a control parameter, leads to misleading conclusions. Instead, by focusing on the average degree of clusters linked by these shortcuts, we demonstrate a more accurate and coherent framework. This new perspective allows for an optimal alignment of system variables with network parameters, resulting in a remarkable data collapse. Additionally, we introduce a phase diagram that distinctly maps the transitional boundary between large and SW regimes. This visual representation not only solidifies our theoretical findings but also offers a practical tool for researchers in the field to better understand and navigate the complexities of SW networks.

## Methods

### “Regular” and “random” nodes

We assume that the network can be split into two sub-networks: a regular one and a random one. Our approach is founded on this assumption.

**Figure 5:**
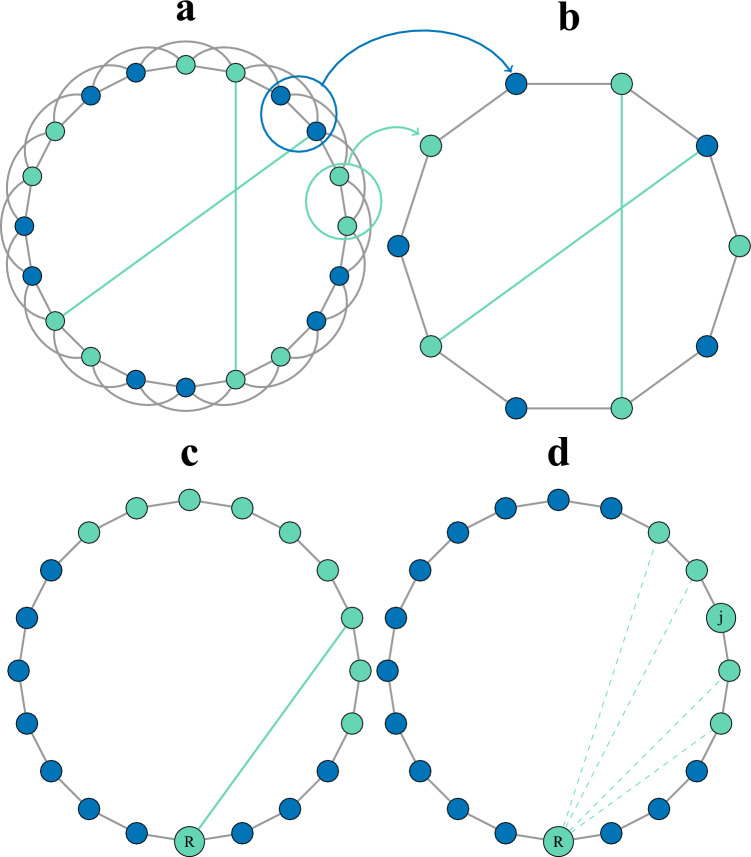
Illustrations explaining the method. The RG transformation of a network (**a**) with $n=20$ and $k=2$ (**a**) to another with $\hat{n}=10$ and $\hat{k}=1$ (**b**). (**c**), distance to the root node *R* after introducing a shortcut. Green nodes whose distance to *R* is changed are called random nodes. Blue nodes whose distance to *R* remain unchanged are called regular nodes. (**d**), represents the case $\{1,i-1\}$, where the green nodes are the positions that cannot be occupied by the intermediate node. Since $\hat{n}=20$ and $i=3$, the number of possible positions for the intermediate node is $\hat{n}-2i=14$.

First, we study in detail the number of neighbors $n_{\ell }$ located at a distance $\ell$ from an arbitrary node by applying the RG in real space on the network (Fig. 5a and b. As the NW model combines both regularity and randomness, we can categorize nodes into two groups: regular nodes $n_{re}(\ell )$ and random nodes $n_{ra}(\ell )$ based on their distance $\ell$ to a randomly selected root node. Regular nodes have not been impacted by the introduction of shortcuts, while random nodes have been affected by shortcuts and their distances have consequently been altered (Fig. 5c).

In the RG *n* becomes $\hat{n}$ $=\frac{n}{k}$ and *k* becomes $\hat{k}=1$ (Fig. 5). Each set of *k* neighboring nodes is replaced by single entity named cluster. The number of clusters in the network is then $\hat{n}$. After adding shortcuts, the probability that a cluster is randomly linked to another cluster is $\hat{\phi }=1- \big (1-\frac{2k\phi }{n}\big )^{k^2}$, for $n\gg k\phi$ we have $\hat{\phi }\approx \frac{2k^3\phi }{n}$. Let $P_{re}(\hat{\ell })$ be the probability that the distance $\hat{\ell }$ between any cluster and the root node has not been changed after adding the shortcuts, and let $P_{ra}(\hat{\ell })$ be the probability that the distance between any cluster and the root node becomes $\hat{\ell }$ after adding the shortcuts (see Fig. 5d). Reducing the distance of a given cluster to the root can be achieved by one, two or more shortcuts. We denote $\pi ^{(M)}(i)$ the probability that the regular initial distance $\hat{\ell }$ of a cluster is not changed to a specific smaller distance *i* through *M* shortcuts. A distinction is made between the following cases:

###  Shortening distances via single shortcuts

Let $\pi ^{(1)}(i)$ the probability that a cluster *j* does not change its initial distance $\hat{\ell }$ to the distance *i* ($i<\hat{\ell }$) through a single shortcut:

7 $$\begin{aligned} \pi ^{(1)}(1)= & {} (1-\hat{\phi }), \nonumber \\ \pi ^{(1)}(2)= & {} (1-\hat{\phi })^4,\nonumber \\ \pi ^{(1)}(3)= & {} (1-\hat{\phi })^{4\cdot 2},\nonumber \\ \vdots \nonumber \\ \pi ^{(1)}(i)= & {} (1-\hat{\phi })^{4(i-1)}. \end{aligned}$$

The preceding expressions follow from the fact that the number of possibilities (jumps) to build a path such as $\hat{\ell }=i$ is $4(i-1)$. In general, this value tends to be overestimated because some of the possibilities correspond to distances $\hat{\ell }<i$, which have already been included in the count for shorter distances. However when the number of jumps is small compared to the size of the network, the expression $4(i-1)$ is exact.

From Eq. (7) we deduce the probability $P^{(1)}_{re}(\hat{\ell })$ that a cluster’s distance $\hat{\ell }$ remains unchanged with a single shortcut:

8 $$\begin{aligned} P_{re}^{(1)}(\hat{\ell })= & {} \pi ^{(1)}(1)\pi ^{(1)}(2)...\pi ^{(1)}(\hat{\ell }-1)\nonumber \\= & {} (1-\hat{\phi })(1-\hat{\phi })^4(1-\hat{\phi })^{4\cdot 2}...(1-\hat{\phi })^{4(\hat{\ell }-2)}\nonumber \\= & {} (1-\hat{\phi })^{1+4+4\cdot 2+4\cdot 3...4(\hat{\ell }-2)}\nonumber \\= & {} (1-\hat{\phi })^{1+4\sum _{i=1}^{\hat{\ell }-1}(i-1)}. \end{aligned}$$

$P_{re}^{(1)}(\hat{\ell })$ can be written:

9 $$\begin{aligned} P_{re}^{(1)}(\hat{\ell })=(1-\hat{\phi })^{4\sum _{i=1}^{\hat{\ell }-1}(i-1)}, \end{aligned}$$

where the term $\pi ^{(1)}(1)=(1-\hat{\phi })$ was omitted^18^.

###  Shortening distances via two shortcuts

Assuming that *R* is the root cluster and *j* is any other cluster in the network, we can determine the number of possible routes between them given that there are two shortcuts connecting the two clusters. To do this, we introduce an arbitrary cluster, denoted as *z*, which lies between the two shortcuts. If the distance between *R* and *j* through *z* is *i*, then the number of possible routes is $i-1$. Specifically, this includes cases such as $\{\{1,i-1\}\{2,i-2\},\cdots ,\{i-1,1\}\}$. For example, if $i=4$, we have three cases: $\{\{1,3\},\{2,2\},\{3,1\}\}$. The first case, $\{1,3\}$, indicates that the distance between cluster *R* and intermediate cluster *z* is 1, and the distance between *z* and cluster *j* is 3. The particular case of $\{1,i-1\}$ can be illustrated as follows:
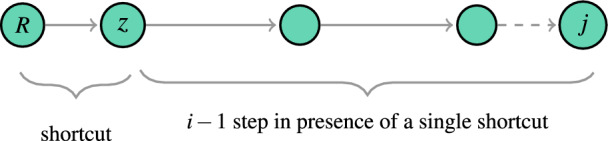


In this case the number of possible paths between *R* and *z* is 1 because they are directly linked with a shortcut, and the number of possible paths between *z* and *j* is $4 (i-2)$ since the distance between them is $i-1$ (see the case of a single shortcut). The probability that the distance between clusters *R* and *j* is not equal to *i* is then $(1-\hat{\phi }^2)^{4(i-2)}$. To make the computations easier, we make an assumption that the probability of all other cases $\{\{2,i-2 \}, \cdots , \{i-1,1\}\}$ is equal to the probability of the case $\{1,i-1\}$, which is a simplified approximation similar to mean field type. As there are $i-1$ such cases, the probability of a cluster not changing its initial distance to the distance *i* through a specific intermediate node is given by $(1-\hat{\phi }^2)^{4(i-2)\times (i-1)}$. The number of possible positions of the intermediate cluster *z* in the network is $\hat{n}-2i$ (Fig. 5d), then the probability that a cluster does not change its initial distance to the distance *i* through two shortcuts is

10 $$\begin{aligned} \pi ^{(2)}(i)=(1-\hat{\phi }^2)^{4(i-1)(i-2)(\hat{n}-2i)}, \end{aligned}$$

whence

11 $$\begin{aligned} P_{re}^{(2)}(\hat{\ell })= & {} \pi ^{(2)}(1)\pi ^{(2)}(2)...\pi ^{(2)}(\hat{\ell }-1)\nonumber \\= & {} (1-\hat{\phi }^2)^{4\sum _{i=1}^{i=\hat{\ell }-1}((i-1)(i-2)(\hat{n}-2i))}, \end{aligned}$$

the term $\pi ^{(2)}(2)$ is excluded from the sum as explained in the case of a single shortcut^18^.

### Shortening distances via *M* shortcuts

By following the same approach as the previous cases, it is easy to generalize for the case of *M* shortcuts. In this case we introduce $M-1$ intermediate clusters, and we get:

12 $$\begin{aligned} \pi ^{(M)}(i)=(1-\hat{\phi }^M)^{4\frac{(i-1)(i-2)\ldots (i-M)}{(M-1)!}(\hat{n}-2i)^{(M-1)}}, \end{aligned}$$

then the probability that a cluster does not change its initial distance through *M* shortcuts is:

13 $$\begin{aligned} P_{re}^{(M)}(\hat{\ell })= & {} \pi ^{(M)}(1)\pi ^{(M)}(2)...\pi ^{(M)}(\hat{\ell }-1)\\\nonumber= & {} (1-\hat{\phi }^M)^{4\sum \limits _{i=1}^{\hat{\ell }-1}\left(\frac{(i-1)(i-2)\ldots (i-M)}{(M-1)!}(\hat{n}-2i)^{M-1}\right)}, \end{aligned}$$

since $\hat{\phi }<1$ we can write

14 $$\begin{aligned} P_{re}^{(M)}(\hat{\ell })=e^{-4\hat{\phi }^M\sum \limits _{i=1}^{\hat{\ell }-1}\left(\frac{(i-1)(i-2)\ldots (i-M)}{(M-1)!}(\hat{n}-2i)^{M-1}\right)}. \end{aligned}$$

It follows that the probability $P_{re}(\hat{\ell })$ for a cluster not changing its initial distance $\hat{\ell }$ after adding any number of shortcuts is:

15 $$\begin{aligned} \nonumber P_{re}(\hat{\ell })= & {} P^{(1)}_{re}(\hat{\ell })P^{(2)}_{re}(\hat{\ell })P^{(3)}_{re}(\hat{\ell })\ldots P^{(\hat{\ell }-1)}_{re}(\hat{\ell })\\\nonumber= & {} e^{-4\hat{\phi }\sum \limits _{i=1}^{\hat{\ell }-1}(i-1)B(i)}, \\ \end{aligned}$$

with

16 $$\begin{aligned} B(i)= & {} 1+[\hat{\phi }(i-2)(\hat{n}-2i)]+[\hat{\phi }^2\frac{(i-2)(i-3)}{2!}(\hat{n}-2i)^{2}]\nonumber \\ \nonumber{} & {} +\ldots +[\hat{\phi }^{i-2}(\hat{n}-2i)^{i-2}]\nonumber \\ \nonumber= & {} 1+[\hat{\phi }(i-2)(\hat{n}-2i)]+[\hat{\phi }^2\frac{(i-2)(i-3)}{2!}(\hat{n}-2i)^{2}]\nonumber \\ \nonumber{} & {} +\ldots +[\hat{\phi }^{i-2}(\hat{n}-2i)^{i-2}]\\ \nonumber= & {} \sum _{j=1}^{i-2}C_j^{i-2}[\hat{\phi }(\hat{n}-2i)]^j1^{i-2-j}\\= & {} (\hat{\phi }(\hat{n}-2i)+1)^{i-2}, \end{aligned}$$

where the last line is deduced from the binomial formula.

Finally we get:

17 $$\begin{aligned} P_{re}(\hat{\ell })= & {} e^{-4\hat{\phi }\sum _{i=1}^{\hat{\ell }-1}(i-1)(\hat{\phi }(\hat{n}-2i)+1)^{i-2}}\\\nonumber\approx & {} e^{-4\hat{\phi }\int _{i=1}^{\hat{\ell }-1}(i-1)(\hat{\phi }(\hat{n}-2i)+1)^{i-2}di}, \end{aligned}$$

where the sum in the exponential is approximated by an integral since in a regular SW network $\hat{\ell } \propto n \gg 1$.

Let $P_{ra}(\hat{\ell })$ be the probability that, due to shortcuts, the distance of any given cluster to a root cluster has changed from its regular distance to a specific distance $\hat{\ell }$. $P_{ra}(\hat{\ell })$ can be written as the product of the probability that this cluster does not change its distance to another strictly less than $\hat{\ell }$ by the probability that the cluster changes its distance to a distance less than or equal to $\hat{\ell }$:

18 $$\begin{aligned} \nonumber P_{ra}(\hat{\ell })= & {} P_{re}(\hat{\ell }-1)(1-\pi _t(\hat{\ell }))\\\nonumber= & {} P_{re}(\hat{\ell }-1)-\pi _t(\hat{\ell })P_{re}(\hat{\ell }-1)\\\nonumber= & {} P_{re}(\hat{\ell }-1)-P_{re}(\hat{\ell })\\\nonumber= & {} -\frac{\partial P_{re}(\hat{\ell })}{\partial \hat{\ell }}\\\nonumber= & {} 4p(\hat{\ell }-2)(p(\hat{n}-2(\hat{\ell }-1)-1))+1)^{\hat{\ell }-3}P_{re}(\hat{\ell })\\= & {} 4p(\hat{\ell }-1)(p(\hat{n}-2\hat{\ell }-1))+1)^{\hat{\ell }-2}P_{re}(\hat{\ell }). \end{aligned}$$

The number of regular clusters after renormalization, $\hat{n}_{re}(\hat{\ell })$, is:

19 $$\begin{aligned} \hat{n}_{re}(\hat{\ell })=2P_{re}(\hat{\ell }), \end{aligned}$$

since when $\hat{k}=1$ each cluster has two neighbors at distance $\hat{\ell }$. On the other hand, the number of random clusters is:

20 $$\begin{aligned} \hat{n}_{ra}(\hat{\ell })=(\hat{n}-2\hat{\ell })P_{ra}(\hat{\ell }), \end{aligned}$$

where $\hat{n}-2\hat{\ell }$ represents the maximum number of clusters with distance (to the root cluster) bigger than $\hat{\ell }$.

Then, the total number of clusters at distance $\ell$ is:

21 $$\begin{aligned} \nonumber \hat{n}(\hat{\ell })= & {} \hat{n}_{re}(\hat{\ell })+\hat{n}_{ra}(\hat{\ell })\\\nonumber= & {} 2P_{re}(\hat{\ell })+(\hat{n}-2\hat{\ell })P_{ra}(\hat{\ell })\\\nonumber= & {} 2P_{re}(\hat{\ell })+(\hat{n}-2\hat{\ell })P_{re}(\hat{\ell }-1)-(\hat{n}-2\hat{\ell })P_{re}(\hat{\ell })\\\nonumber= & {} (\hat{n}-2\hat{\ell })P_{re}(\hat{\ell }-1)-(\hat{n}-2(\hat{\ell }+1))P_{re}(\hat{\ell })\\\nonumber= & {} v(\hat{\ell }-1)-v(\hat{\ell }),\\ \end{aligned}$$

with $$v(\hat{\ell })=(\hat{n}-2(\hat{\ell }+1))P_{re}(\hat{\ell })$$.

In order to measure the impact of individual sub-networks, we determine the total number of clusters within each sub-network, which we refer to as $\hat{S}_{re}$ and $\hat{S}_{ra}$:

22 $$\begin{aligned} \nonumber \hat{S}_{re}= & {} \hat{n}_{re}(1)+\sum ^{\frac{\hat{n}}{2}}_{\hat{\ell }=2}\hat{n}_{re}(\hat{\ell })\\\nonumber= & {} \hat{n}_{re}(1)+\int ^{\frac{\hat{n}}{2}}_2\hat{n}_{re}(\hat{\ell })d\hat{\ell }\\\nonumber= & {} \hat{n}_{re}(1)+2\int ^{\frac{\hat{n}}{2}}_2e^{-4\hat{\phi }\int _{j=1}^{\hat{\ell }-1}(j-1)(\hat{\phi }(\hat{n}-2j)+1)^{j-2}dj}d\hat{\ell },\\ \end{aligned}$$

which becomes for $\hat{n}\gg 1$:

23 $$\begin{aligned} \nonumber \hat{S}_{re}\approx & {} \hat{n}_{re}(1)+2\int ^{\frac{\hat{n}}{2}}_2e^{-4\hat{\phi }\int _{j=1}^{\hat{\ell }}jdj}d\hat{\ell }\\\nonumber\approx & {} \hat{n}_{re}(1)+2\int ^{\frac{\hat{n}}{2}}_2e^{-2\hat{\phi }\hat{\ell }^2}d\hat{\ell }\\\nonumber\approx & {} 2+2\Big [\frac{\sqrt{\frac{\pi }{2}}erf(\sqrt{2\hat{\phi }}\hat{\ell })}{2\sqrt{\hat{\phi }}}\Big ]^{\frac{\hat{n}}{2}}_2 \hspace{1cm} (\hat{n}_{re}(1)=2) \\\approx & {} 2+ 2\sqrt{\frac{\pi }{8\hat{\phi }}}\Big [erf(\sqrt{2\hat{\phi }}\frac{\hat{n}}{2})-erf(2\sqrt{2\hat{\phi }})\Big ]. \end{aligned}$$

When regularity is dominating $\hat{\phi }\ll 1$, then $erf(2\sqrt{2\hat{\phi }}) \approx \dfrac{2}{\sqrt{\pi }}2\sqrt{2\hat{\phi }}$, we get:

24 $$\begin{aligned} \nonumber \hat{S}_{re}\approx & {} 2+ 2\sqrt{\frac{\pi }{8\hat{\phi }}}\Big [erf(\sqrt{2\hat{\phi }}\frac{\hat{n}}{2})-\dfrac{2}{\sqrt{\pi }}2\sqrt{2\hat{\phi }}\Big ] \\\nonumber\approx & {} 2+2\sqrt{\frac{\pi }{8\hat{\phi }}}erf(\sqrt{\frac{\hat{\phi }\hat{n}^2}{2}})-4 \\\nonumber\approx & {} \hat{n}\Big (\sqrt{\frac{\pi }{2\hat{\phi }\hat{n}^2}}erf(\sqrt{\frac{\hat{\phi }\hat{n}^2}{2}})-\frac{2}{\hat{n}}\Big ) \\\approx & {} \hat{n}\sqrt{\frac{\pi }{2\hat{\phi }\hat{n}^2}}erf(\sqrt{\frac{\hat{\phi }\hat{n}^2}{2}}). \end{aligned}$$

While $\hat{\phi }=\frac{2k^3\phi }{n}$ and $\hat{n}=\frac{n}{k}$ so $\frac{\hat{\phi }\hat{n}^2}{2}=kn\phi$, which is none other than the mean number of shortcuts in the network. The sum of clusters in the regular sub-network is then:

25 $$\begin{aligned} \hat{S}_{re}=\hat{n}(1-h(kn\phi )), \end{aligned}$$

with $$h(x)=1-\sqrt{\frac{\pi }{4x}}erf(\sqrt{x})$$, and $$x=kn\phi$$.

The number of clusters in the random sub-network is deduced from:

26 $$\begin{aligned} \nonumber \hat{S}_{ra}\approx & {} \hat{n}-\hat{S}_{re}\\\nonumber\approx & {} \hat{n}-\hat{n}\sqrt{\frac{\pi }{2\hat{\phi }\hat{n}^2}}erf\left(\sqrt{\frac{\hat{\phi }\hat{n}^2}{2}}\right) \\\nonumber\approx & {} \hat{n}\Bigg (1-\sqrt{\frac{\pi }{2\hat{\phi }\hat{n}^2}}erf\left(\sqrt{\frac{\hat{\phi }\hat{n}^2}{2}}\right)\Bigg ) \\\approx & {} \hat{n}h(x). \end{aligned}$$

Since each cluster is made up with *k* nodes, the total number of regular nodes is $S_{re}=n(1-h(x))$ and the total number of random nodes is $S_{ra}=nh(x)$.

### “Regular” and “random” mean distance

The mean distance in the network is $\nonumber \langle \hat{\ell } \rangle =\nonumber \langle \hat{\ell }_{re} \rangle + \nonumber \langle \hat{\ell }_{ra} \rangle$, where $\langle \hat{\ell }_{re} \rangle$ is the mean distance in the regular sub-network, and $\langle \hat{\ell }_{ra} \rangle$ is the mean distance in the random sub-network.

$\langle \hat{\ell }_{re} \rangle$ is deduced from

27 $$\begin{aligned} \langle \hat{\ell }_{re} \rangle =\frac{\int _1^{\frac{\hat{n}}{2}}\hat{\ell }\cdot \hat{n}_{re}(\hat{\ell })d\hat{\ell }}{\hat{n}}, \end{aligned}$$

using (17) and (19) we have

$$\begin{aligned} \langle \hat{\ell }_{re} \rangle= & {} \frac{1}{\hat{n}}\int ^{\frac{\hat{n}}{2}}_1 2\hat{\ell }e^{-4\hat{\phi }\int _{j=1}^{\hat{\ell }-1}(j-1)(\hat{\phi }(\hat{n}-2j)+1)^{j-2}dj}d\hat{\ell }, \end{aligned}$$

taking $\hat{\phi }$ small and considering $\hat{\ell }=\mathcal {O}(n)$ (regular network), we get

$$\begin{aligned} \int ^{\frac{\hat{n}}{2}}_2 2\hat{\ell }e^{-4\hat{\phi }\int _{j=1}^{\hat{\ell }-1}(j-1)(\hat{\phi }(\hat{n}-2j)+1)^{j-2}dj}d\hat{\ell }\approx \int _1^{\frac{\hat{n}}{2}}2\hat{\ell }e^{-2\hat{\phi }\hat{\ell }^2}d\hat{\ell }, \end{aligned}$$

then

28 $$\begin{aligned} \langle \hat{\ell }_{re} \rangle\approx & {} \frac{1}{\hat{n}}\int _1^{\frac{\hat{n}}{2}}2\hat{\ell }e^{-2\hat{\phi }\hat{\ell }^2}d\hat{\ell } \nonumber \\\approx & {} \frac{1}{\hat{n}}\frac{e^{-2\hat{\phi }}-e^{-\frac{\hat{\phi }\hat{n}^2}{2}}}{2\hat{\phi }}\nonumber \\\approx & {} \frac{\hat{n}}{4}\frac{1-e^{-\frac{\hat{\phi }\hat{n}^2}{2}}}{\frac{\hat{\phi }\hat{n}^2}{2}}. \end{aligned}$$

$\langle \hat{\ell }_{re} \rangle$ can then be written in the following form:

29 $$\begin{aligned} \langle \hat{\ell }_{re} \rangle\approx & {} \hat{n}\frac{1-e^{-x}}{4x}. \end{aligned}$$

$\langle \hat{\ell }_{ra}\rangle$ can deduced from the maximum of $\hat{n}_{ra}(\hat{\ell })$ as explained in^19^. Explicitly, we have to solve $\frac{\partial \hat{n}_{ra}(\hat{\ell })}{d\hat{\ell }}=0$. From (17) and (18) we obtain:

30 $$\begin{aligned} P_{ra}(\hat{\ell })=u(\hat{\ell })e^{-\int _{j=1}^{\hat{\ell }-1}u(j)dj}, \end{aligned}$$

where $u(\hat{\ell })=4\hat{\phi }(\hat{\ell }-1)(\hat{\phi }(\hat{n}-2\hat{\ell })+1)^{\hat{\ell }-2}$. When shortcuts are present, the mean distance in the network is considerably lowered, we can therefore consider $\hat{n}-2\hat{\ell }\approx \hat{n}$, then

31 $$\begin{aligned} u(\hat{\ell })=4\hat{\phi }(\hat{\ell }-1)(y+1)^{\hat{\ell }-2}, \end{aligned}$$

where $y=\hat{\phi }\hat{n}$ is the mean degree of clusters linked by shortcuts. It is worth noting that *y* is analogous to the mean degree of nodes in the Erdös-Rényi network. The number of random clusters becomes:

32 $$\begin{aligned} \hat{n}_{ra}(\hat{\ell })=\hat{n}u(\hat{\ell })e^{-\int _{j=1}^{\hat{\ell }-1}u(j)dj}, \end{aligned}$$

then

33 $$\begin{aligned} \nonumber \frac{\partial \hat{n}_{ra}(\hat{\ell })}{\partial \hat{\ell }}= & {} \hat{n}\frac{\partial u(\hat{\ell })}{\partial \hat{\ell }}e^{-\int _{j=1}^{\hat{\ell }-1}u(j)dj}+\hat{n}u(\hat{\ell })\frac{\partial e^{-\int _{j=1}^{\hat{\ell }-1}u(j)dj}}{\partial \hat{\ell }}\\\nonumber= & {} \hat{n}\frac{\partial u(\hat{\ell })}{\partial \hat{\ell }}e^{-\int _{j=1}^{\hat{\ell }-1}u(j)dj}-\hat{n}u(\hat{\ell })^2e^{-\int _{j=1}^{\hat{\ell }-1}u(j)dj}.\\ \end{aligned}$$

The maximum of $\hat{n}_{ra}(\hat{\ell })$ is then given by the solution of

34 $$\begin{aligned} \frac{\partial u(\hat{\ell })}{\partial \hat{\ell }}-u(\hat{\ell })^2=0. \end{aligned}$$

From (31) we get $\frac{\partial u(\hat{\ell })}{\partial \hat{\ell }}=u(\hat{\ell })\big [\frac{1}{\hat{\ell }-1}+\ln (y+1)\big ]$. Since $y=\hat{\phi }\hat{n}=2k^2\phi$ does not depend on the size of the network $\hat{n}$, whereas $\hat{\ell }$ increases with $\hat{n}$, so we neglect $\frac{1}{\hat{\ell }-1}$, and obtain $\frac{\partial u(\hat{\ell })}{\partial \hat{\ell }}=u(\hat{\ell })\ln (y+1)$. Replacing in (34), we get:

35 $$\begin{aligned} u(\hat{\ell })=\ln (y+1). \end{aligned}$$

From (31) we get the distance $\hat{\ell }_{max}$ at which $\hat{n}_{ra}(\hat{\ell })$ is maximum:

36 $$\begin{aligned} \hat{\ell }_{max}=\frac{W\big (\frac{(\ln (y+1))^2(y+1)}{4\hat{\phi }}\big )}{\ln (y+1)}+1, \end{aligned}$$

with *W*(*x*) is the Lambert function.

Multiplying $\hat{\ell }_{max}$ by the fraction of random nodes, $h(x)=1-\sqrt{\frac{\pi }{4x}}{{\,\textrm{erf}\,}}(\sqrt{x})$, we get the mean distance in the random network:

37 $$\begin{aligned} \langle \hat{\ell }_{ra} \rangle =\Bigg (\frac{W\bigg (\frac{\left(\ln (y+1)\right)^2(y+1)}{4\hat{\phi }}\bigg )}{\ln (y+1)}+1\Bigg )h(x). \end{aligned}$$

## Data Availability

The datasets used and/or analyzed during the current study are available upon reasonable request from the corresponding author.
